# In Situ Construction of Superhydrophobic Photothermal Coatings Based on Metal–Polyphenol Coordination Complex for Anti-/De-Icing Applications

**DOI:** 10.3390/polym18111286

**Published:** 2026-05-24

**Authors:** Zhiheng Zhao, Buyu Luo, Guoliang Chen, Tianbao Zhao, Yifei Chen, Zhengping Zhao, Baoshu Chen

**Affiliations:** 1 School of Materials Science and Engineering, Xihua University, Chengdu 610039, China; zhaozh123@xhu.edu.cn (Z.Z.); xhucgl@icloud.com (G.C.); 0120140042@xhu.edu.cn (T.Z.); 2 The State Key Laboratory of Polymer Materials Engineering, Polymer Research Institute of Sichuan University, Chengdu 610065, China; 3 Zhijiang College, Zhejiang University of Technology, Hangzhou 310014, China

**Keywords:** photothermal, superhydrophobic, coating, anti-icing, de-icing, UV protection

## Abstract

Superhydrophobic photothermal coatings have great potential in anti-icing and de-icing applications. However, how to construct superhydrophobic coatings with high photothermal conversion performance and an appropriate rough structure is still a challenge. In this study, we first constructed the photothermal nanosphere coating by in situ co-deposition of tannic acid (TA) and (3-aminopropyl) triethoxysilane (APTES) and then by the coordination of iron ions (Fe^3+^). A superhydrophobic photothermal coating with a micro–nano–nano hierarchical rough structure was constructed by further applying a polydimethylsiloxane (PDMS)/hydrophobic fumed silica (SiO_2_) coating. The coating has excellent superhydrophobic (water contact angle (WCA) of 158°) and efficient photothermal conversion performance (75 °C). Based on this, the coated fabric shows ideal performance in passive anti-icing and active de-icing tests. At the same time, the coated fabric also has an ideal UV shielding effect, which can ensure the long-term and efficient operation of the coated fabric in the outdoor sunlight. This preparation strategy provides an innovative method for the development of superhydrophobic photothermal coating materials and has broad application prospects in the field of flexible anti-/de-icing applications.

## 1. Introduction

Ice formation and accretion on the surfaces of outdoor equipment not only alter the intrinsic physical and structural properties of substrate materials but also trigger a series of severe safety hazards and substantial economic losses in practical applications [1,2,3]. To address this intractable icing issue, a variety of anti-icing and de-icing strategies have been proposed to date [4,5,6,7]. While these approaches achieve a certain degree of icing control efficacy, they suffer from inherent drawbacks including high energy consumption, secondary environmental pollution, intricate operation procedures and potential damage to substrate materials, which greatly restrict their long-term and effective large-scale application [8,9]. Thus, the development of high-efficiency, low-energy-consumption and eco-friendly anti-icing/de-icing materials has emerged as a research hotspot in the fields of surface engineering and materials science.

Inspired by the natural lotus leaf effect, superhydrophobic surfaces derive their unique anti-wetting performance from the synergistic effect of hierarchical micro–nanoscale rough structures and low-surface-energy chemical components [10]. Such surfaces exhibit distinctive application advantages in the field of passive anti-icing: they can trap a large volume of air to form a stable air cushion at the solid–liquid interface, which reduces the actual contact area between water droplets and the surface, remarkably diminishes liquid adhesion to the surface and simultaneously hinders interfacial heat transfer between water and the substrate [11,12,13]. This characteristic not only facilitates the rolling off of water droplets from the surface prior to freezing but also prolongs the freezing time of water droplets and reduces the ice adhesion strength after freezing, thereby realizing a passive anti-icing effect of retarding water freezing and mitigating ice adhesion [14]. In addition, superhydrophobic surfaces possess excellent self-cleaning performance, which can prevent the accumulation of dust, moisture and other icing-promoting substances on the surface and further enhance their long-term passive anti-icing stability in complex outdoor environments [15,16,17].

However, the sole passive anti-icing function of superhydrophobic surfaces is insufficient to meet the practical anti-icing/de-icing demands in complex and harsh low-temperature environments, particularly the requirement for the efficient removal of accumulated ice on material surfaces [18,19]. In recent years, the integration of superhydrophobicity with photothermal conversion capability has broken the limitations of single-functional surfaces and emerged as a novel and high-efficiency anti-icing/de-icing strategy, enabling the synergistic effect of passive anti-icing and active photothermal de-icing [20,21]. On the one hand, the superhydrophobic property of the multifunctional composite surface still exerts its inherent passive anti-icing advantages, retarding the freezing of water droplets and reducing the adhesion of ice crystals. On the other hand, the photothermal conversion component can efficiently convert solar energy into thermal energy, rapidly elevating the material surface temperature to above the freezing point of water, thus realizing the active in situ melting of accumulated ice under solar irradiation [22,23,24]. Meanwhile, the superhydrophobic surface allows the melted water to roll off and flow away rapidly, which effectively avoids the re-icing phenomenon caused by secondary freezing and further improves the efficiency and durability of photothermal de-icing [25,26]. Such superhydrophobic photothermal functional surfaces combine low-energy-consumption passive anti-icing with solar-driven active de-icing, thereby exhibiting broad application prospects in relevant engineering fields.

In this work, a novel strategy for the in situ construction of superhydrophobic photothermal coatings based on metal–polyphenol coordination complex is proposed. Specifically, a dense nanosphere layer is first grown in situ on microscale cotton fibers via the reaction of tannic acid (TA) and 3-aminopropyltriethoxysilane (APTES) to construct a micro–nanoscale rough structure, and the coordination reaction between TA and Fe^3+^ ions is utilized to endow the coating with excellent photothermal conversion properties. Subsequently, a composite layer of polydimethylsiloxane (PDMS) with low surface energy and hydrophobic fumed silica (SiO_2_) is coated on the coating surface: PDMS provides favorable interfacial adhesion performance, while the introduction of SiO_2_ enables the construction of a micro–nano–nano hierarchical rough structure. The coated fabric exhibits outstanding superhydrophobic and photothermal conversion performances. Based on these superior properties, the coated fabric can significantly prolong the freezing time of water and rapidly remove ice from its surface under light-driven conditions. Furthermore, the superhydrophobic photothermal coating demonstrates an ideal UV shielding effect, which can provide sufficient protection to ensure the long-term and high-efficiency operation of the coated fabric under outdoor solar irradiation. We anticipate that such a superhydrophobic photothermal coating will possess excellent application potential in the field of flexible anti-icing/de-icing.

## 2. Experimental

### 2.1. Materials

Tannic acid (TA, 95%), (3-aminopropyl) triethoxysilane (APTES, 98%), Tris (hydroxymethyl) aminomethane and ferric sulfate (Fe_2_(SO_4_)_3_, 99%+) were supplied by Adamas Reagent, Ltd. (Shanghai, China). Hydrophobic fumed silica (SiO_2_, AEROSIL R812S, treated hexamethyl disilazane (HMDS); average particle size: 7 nm) was purchased from Evonik Industries AG (Essen, Germany). Polydimethylsiloxane (PDMS, Sylgard 184A) and curing agent (Sylgard 184B) were purchased from Dow Corning Corporation (Midland, MI, USA). Cotton fabric (CF) was obtained from the Yongsheng Textile Co., Ltd. (Shijiazhuang, China). (weight 110 g/m^2^). All reagents were used as received without further purification.

### 2.2. Preparation of Superhydrophobic Photothermal Coated Fabric

#### 2.2.1. Preparation of T@CF

In total, 60 mg of tannic acid (TA) was dissolved in 25 mL of Tris buffer (pH = 8.5), and then 60 mg of APTES dissolved in 5 mL of anhydrous ethanol was added to the mixture. A pre-washed cotton fabric (5 cm × 5 cm) was immersed in the resulting solution and stirred at room temperature for 12 h. This yielded a CF coated with TA-APTES nanospheres (T@CF).

#### 2.2.2. Preparation of FT@CF

T@CF was immersed in an aqueous solution of Fe_2_(SO_4_)_3_ (10 mg/mL, 50 mL) at room temperature for 1 h, then thoroughly rinsed with deionized water to remove residual ions, and dried in an oven at 60 °C. The resulting Fe-chelated sample was named FT@CF.

#### 2.2.3. Preparation of PSFT@CF

PDMS (0.5 wt%) was added with hydrophobic fumed silica (SiO_2_, 0.5 wt%) in ethyl acetate and sonicated for 1 h. Subsequently, the curing agent was added in a 10:1 mass ratio (PDMS: curing agent) and stirred for 10 min. Then, the FT@CF was immersed in the dispersion for 10 min and dried at 60 °C for 5 h. The obtained sample was named PSFT@CF. For comparison, CF and FT@CF treated with only 0.5 wt% PDMS were prepared. The resulting samples were named P@CF and PFT@CF, respectively.

### 2.3. Characterizations

The microstructures of the samples were observed using field-emission scanning electron microscopy (FE-SEM, NovaNano450, FEI, Hillsboro, OR, USA) at an accelerating voltage of 5 kV. The surface chemical compositions of the samples were determined through an X-ray photoelectron spectroscopy (XPS, XSAM800, Kratos, Manchester, UK) with an Al Kα X-ray source (h*υ* = 1486.6 eV). Water contact angle (WCA) was measured with an optical contact angle measuring device (DSA25S, Krüss, Hamburg, Germany) with a 5.0 μL drop, five measurements were taken at different positions on each sample, and the average value was calculated.

The UV-vis-NIR reflectivity and transmissivity of samples were measured by a UV-vis-NIR spectrometer (UV-3600i Plus, SHIMADZU, Kyoto, Japan). The absorption (α) of different samples in the solar radiation band of 300–2500 nm was calculated using the following equation:

$$\alpha =\frac{\int_{300}^{2500}\alpha (\lambda )i(\lambda )d(\lambda )}{\int_{300}^{2500}i(\lambda )d(\lambda )}$$

Here, α(λ) is the spectral absorptance obtained from the equation α=1−r−t (where α, *r* and *t* represent the spectral absorptance, reflectance and transmittance, respectively). *i*(λ) is the spectral solar irradiance (W m^−2^ nm^−1^) obtained in accordance with the ASTM G 173-03 standard.

The ultraviolet protection factor (UPF) was measured according to the GB/T18830-2009 standard.

### 2.4. Evaluation of Photothermal Performance

The samples (4 cm × 4 cm) were placed in a polyethylene foam box at room temperature and irradiated under a xenon lamp (CEL-S500-T5, Beijing China Education Au-light Co., Ltd., Beijing, China), which was usually used as a solar simulator. The intensity of simulated sunlight was measured by a solar power meter (CEL-FZ-A, Beijing China Education Au-light Co., Ltd., Beijing, China). A multi-channel temperature monitor (JK808, JINKO, Changzhou, China) and a thermal infrared (IR) camera (346L, FOTRIC, Shanghai, China) were used to display and record the surface temperature of the samples.

### 2.5. Passive Anti-Icing and Active Photothermal De-Icing Experiments

To evaluate anti-icing performance, the freezing time of water droplets on the surface was measured. The samples were placed on a semiconductor cooling stage set to a temperature of −17 °C and a relative humidity of 35 ± 5% to simulate harsh environmental conditions. Subsequently, water droplets (0.15 mL) were deposited onto the sample surface using a pipette, and the freezing process was recorded in situ using a smartphone. The time taken for the water droplet to transition from a liquid to a fully solid state was defined as the freezing time. For comparison, CF treated with only 0.5 wt% PDMS (P@CF) was prepared. The active photothermal de-icing performance of the coating was measured by recording the time it took for the same volume of ice to melt and slip off the coating at one sunlight intensity. First, water was poured into a silicone mold in a cold environment to be frozen into a block. The ice was then placed on the surface of the coating and left in a freezer at −17 °C for an additional 12 h with the relative humidity set to 35 ± 5%. Then, the sample was placed on a semiconductor refrigeration table under simulated sunlight with the light intensity set to one sun.

## 3. Results and Discussion

**Figure 1:**
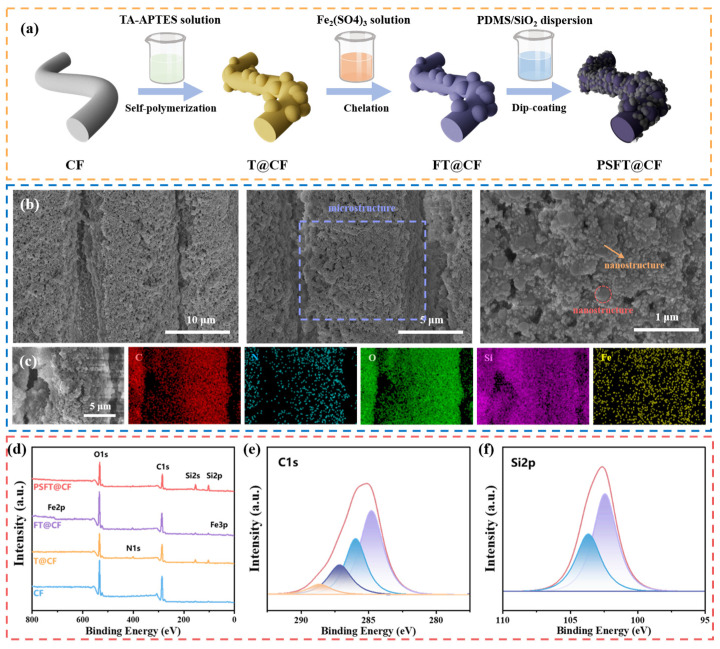
(**a**) Schematic illustration of the fabrication of superhydrophobic photothermal coating. (**b**) SEM and (**c**) EDS elemental mapping images of PSFT@CF. (**d**) XPS spectrum of CF, T@CF, FT@CF and PSFT@CF. (**e**) XPS high-resolution spectra of C 1s of T@CF. (**f**) XPS high-resolution spectra of Si 2p of PSFT@CF.

Figure 1a illustrates the fabrication procedure for the in situ synthesized superhydrophobic photothermal coating based on a metal–polyphenol coordination complex. Under weakly alkaline conditions, the hydrolysis products of APTES form long-chain molecules rich in amino groups through condensation reactions. These molecules react with the oxidation products of TA via Michael addition and Schiff base reactions, generating a large number of nanospheres in the solution mixture. These nanospheres adhere to the surface of the cotton fibers to form the first layer of nano-roughened structure [27,28]. The metal–polyphenol coordination complex is subsequently generated via the coordination reaction between Fe^3+^ and TA (Figure S1). Multiple deposition cycles enhance the loading of TA and iron ions, thereby improving the photothermal conversion performance. Thereafter, the FT@CF sample is modified with PDMS and hydrophobic fumed silica (SiO_2_) to reduce surface energy. SiO_2_ nanoparticles introduce additional nanoscale surface roughness, while PDMS ensures the stable adhesion of SiO_2_ nanoparticles onto the Fe^3+^-TA coordination complex. After coating with TA-APTES nanospheres, the surface color of T@CF changes from white to yellow. Chelation with Fe^3+^ ions further transforms the color to gray-black. Following the transparent PDMS/SiO_2_ coating, the appearance of the PSFT@CF fabric shows minimal change compared to the FT@CF fabric (Figure S2). This fabrication strategy, which integrates micro–nano–nano hierarchical roughness with Fe^3+^-TA coordination complex, ensures the coating achieves a favorable combination of highly efficient photothermal conversion and exceptional superhydrophobicity.

The surface micromorphology of the samples is characterized by scanning electron microscopy (SEM). The CF exhibits a relatively smooth surface (Figure S3a,a’). After the TA-APTES reaction, the surface of T@CF is densely covered with numerous nanoparticles approximately 300 nm in diameter (Figures S3b,b’ and S4). As shown in Figure S3c,c’, the micromorphology of FT@CF after Fe^3+^ chelation shows no obvious difference compared with T@CF. As presented in Figure 1b, following coating with PDMS/SiO_2_, SiO_2_ nanoparticles of around 7 nm are anchored onto the TA-APTES nanospheres via PDMS bonding, verifying the successful construction of hierarchical micro/nano-rough structures. EDS elemental mapping (Figure 1c) reveals that C, N, O, Si, and Fe elements are uniformly distributed on the fiber surface.

Figure S5 shows the ATR-FTIR spectra of CF, T@CF, FT@CF, and PSFT@CF samples. The spectrum of the pristine cotton fabric exhibits characteristic peaks at 3330 cm^−1^ and 2922 cm^−1^, corresponding to the stretching vibrations of -OH and -CH_2_ groups, respectively, as well as two prominent cellulose peaks at 1165 cm^−1^ and 1025 cm^−1^, attributed to the bending vibration of -OH and the stretching vibration of C-O-C [29]. In the spectrum of T@CF, new peaks appear at 1616 cm^−1^ and 1430 cm^−1^, which can be assigned to the stretching vibrations of the aromatic ring, indicating the successful modification of the cotton fabric by tannic acid (TA) [30]. A peak at 1506 cm^−1^, corresponding to N-H bending vibrations, further confirms the formation of a Schiff base between TA and APTES [27]. After chelation with Fe^3+^ ions, the FTIR spectrum of FT@CF shows no significant difference from that of TAC, suggesting that the introduction of Fe^3+^ does not significantly alter the overall structure of the coating. After dip coating, the characteristic peak in the PSFT@CF spectrum that appeared at 795 cm^−1^ was attributed to the symmetric stretching vibration of Si–O–Si in SiO_2_ and PDMS. Furthermore, the appearance of new peaks at 1260 cm^−1^ was attributed to the –CH_3_ symmetric stretching vibration in Si–CH_3_, indicating the existence of PDMS [31]. The absorption bands observed at 2960 cm^−1^ and 845 cm^−1^ should be related to C–H stretching and CH_2_ rocking of the CH_3_ group in PDMS [32]. Figure 1c displays the full XPS spectra of CF, T@CF, FT@CF and PSFT@CF samples. Comparative analysis of the XPS spectra between bare CF and T@CF uncovers the emergence of a novel N 1s characteristic peak at 401.1 eV within the spectrum of T@CF, which directly demonstrates the successful immobilization of APTES onto the cotton fiber substrate [33,34]. Meanwhile, the distinct detection of Si 2s and Si 2p peaks located at 154.5 eV and 103.2 eV, respectively, provides further conclusive evidence for the effective grafting of APTES onto the cotton surface, as documented in the previous literature. With regard to the FT@CF sample, the observation of characteristic Fe 2p and Fe 3p peaks in its XPS spectrum verifies the efficient chelation interaction between Fe^3+^ and TA. For the PSFT@CF, the characteristic peaks assigned to N and Fe elements fade nearly completely, whereas the intensities of Si 2s and Si 2p peaks are remarkably intensified, manifesting that the superhydrophobic PDMS/SiO_2_ coating has been successfully fabricated onto the fiber surface. Furthermore, Figure 1e illustrates the high-resolution C 1s XPS spectrum of T@CF, in which four newly generated peaks centered at 284.7 eV, 285.9 eV, 287.1 eV, and 288.6 eV are ascribed to C-Si, C-N, C=N and C=O chemical bonds, respectively. These spectral findings corroborate the occurrence of Michael addition and Schiff base reactions between TA and APTES, validating the successful co-modification of CF with TA and APTES [35,36]. Correspondingly, Figure 1f exhibits the high-resolution Si 2p XPS spectrum of PSFT@CF, where two characteristic peaks situated at 102.4 eV and 103.6 eV correspond to Si-O and Si-O-Si bonds, respectively. This result further consolidates the successful modification of FT@CF with PDMS and hydrophobic SiO_2_ nanoparticles, thus contributing to the boosted hydrophobic performance of the as-prepared composite coating [37]. Elemental analysis further corroborates the successful fabrication of the superhydrophobic photothermal coating.

**Figure 2:**
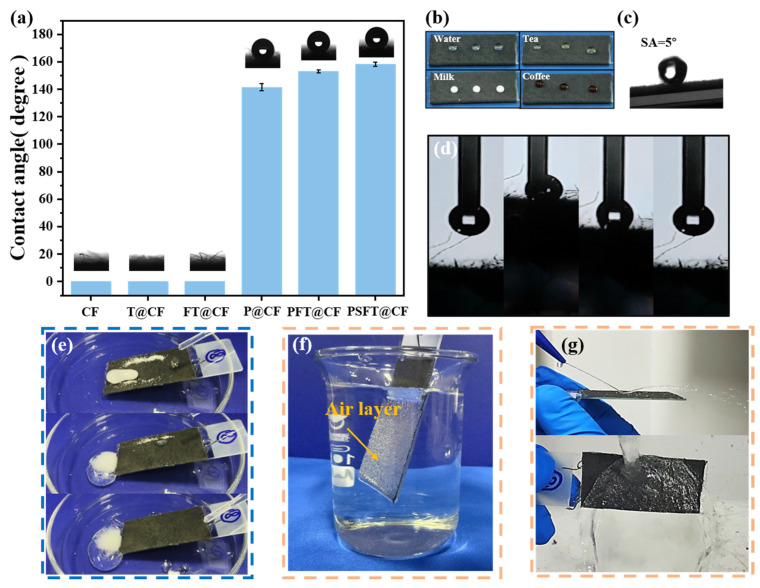
(**a**) WCAs of the samples. (**b**) The optical images of water, tea, milk and coffee on PSFT@CF. (**c**) The slide angle of PSFT@CF. (**d**) Contact and detachment of a water droplet from the coating surface. (**e**) Self-cleaning performance and (**f**) silver mirror-like phenomenon of the PSFT@CF. (**g**) Water jet bounces off the surface of PSFT@CF.

The water contact angle (WCA) variations of different samples are presented in Figure 2a. Pristine CF, T@CF, and FT@CF exhibit superhydrophilicity and can be fully wetted by water, owing to the abundant hydrophilic groups present in the TA-APTES layer. After modification with PDMS alone, P@CF exhibits a hydrophobic surface with a WCA of 141.5° ± 2.5°. The TA-APTES layer introduces nanoscale surface roughness (Figure S6), enabling PFT@CF to achieve superhydrophobicity with a WCA increased to 153°. Furthermore, the incorporation of SiO_2_ nanoparticles further optimizes the surface roughness, resulting in a high WCA of up to 158° for PSFT@CF. When exposed to common daily aqueous solutions, including water, tea, milk, and coffee, PSFT@CF demonstrates excellent liquid-repellent behavior. The four kinds of aqueous droplets remain spherical on the PSFT@CF surface (Figure 2b). Meanwhile, PSFT@CF possesses a remarkably low sliding angle of approximately 5° (Figure 2c). As illustrated in Figure 2d, water droplets readily detach from the coating surface without residual adhesion, indicative of low water adhesion. This phenomenon arises from the formation of a stable air cushion between the water droplets and the coated surface, corresponding to the stable Cassie–Baxter state. The trapped air layer at the solid–liquid interface significantly weakens interfacial interactions by effectively reducing both the contact area and contact duration between water droplets and the coating.

The self-cleaning property of PSFT@CF was also systematically verified. For the self-cleaning test, PSFT@CF was tilted at a certain angle by placing it on the edge of a Petri dish and covered with quartz sand particles. Water droplets rolled down the coating surface rapidly, effectively removing the sand contaminants. No residual debris was observed on the PSFT@CF surface after the self-cleaning test (Figure 2e). Furthermore, a distinct mirror-like phenomenon was observed when the coated fabric was immersed in water, which is attributed to the light reflection of the air layer trapped by the micro/nanostructures (Figure 2f). As depicted in Figure 2g, a water jet impinges onto the coated fabric surface and rebounds rapidly. Meanwhile, no water adhesion or residue is observed on the PSFT@CF surface, demonstrating the robust superhydrophobic stability of the as-prepared coating.

**Figure 3:**
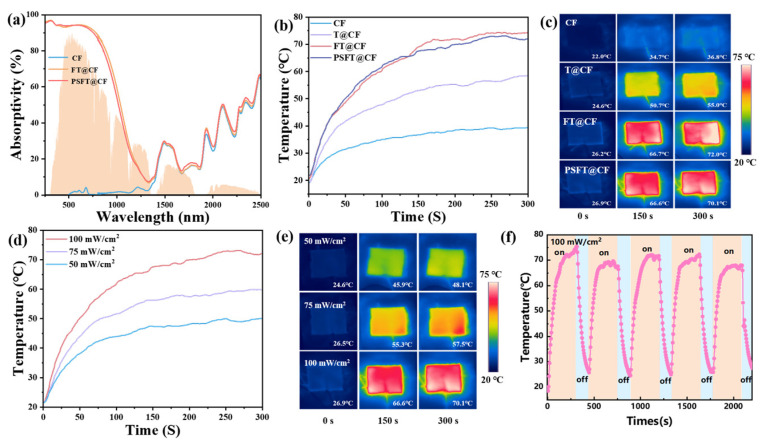
(**a**) UV-vis-NIR absorptivity at 250–2500 nm of CF, FT@CF, and PSFT@CF. (**b**) Temperature variation curves and (**c**) infrared thermal image of CF, T@CF, FT@CF, and PSFT@CF under 100 mW/cm^2^ sun irradiation. (**d**) Temperature variation curves and (**e**) infrared thermal image of the PSFT@CF under different sunlight densities. (**f**) The photothermal conversion of PSFT@CF during 5 heating and cooling cycles (the orange areas indicate when the lighting is on, and the blue areas indicate when the lighting is off).

The reflectance and transmittance of various samples within the solar spectrum were measured using a UV-vis-NIR spectrophotometer, from which the light absorption curves of the samples were derived. The average solar absorption efficiency was further determined via weighted calculation. As illustrated in Figure 3a, the optical absorption profile of FT@CF is distinctly higher than that of CF over the entire solar spectral range (250–2500 nm). FT@CF achieves an average optical absorption of 72.9%, which is attributed to the synergistic broadband light absorption effect of the black photothermal coordination complex formed via the coordination between Fe^3+^ and TA. In comparison with FT@CF, the average optical absorption of PSFT@CF (71.8%) remains nearly unchanged, indicating that the intrinsic optical absorption property of the Fe^3+^-TA-APTES layer is well preserved after coating with the transparent PDMS/SiO_2_ superhydrophobic layer.

To investigate the photothermal conversion performance, the surface temperature evolution of various samples is recorded using a thermocouple under simulated solar irradiation in the laboratory. Upon exposure to 1 sun illumination (100 mW/cm^2^), FT@CF exhibits a rapid temperature increase from 20 °C to approximately 73 °C within 300 s, representing a remarkably enhanced photothermal response relative to CF (39.4 °C) and T@CF (58.3 °C), thus demonstrating the optimal photothermal conversion efficiency (Figure 3b). The photothermal conversion profile of PSFT@CF is almost identical to that of FT@CF, which is consistent with the optical absorption measurements. Furthermore, observations from infrared thermal imaging further validate the superior photothermal conversion characteristics of both FT@CF and PSFT@CF (Figure 3c). Furthermore, the photothermal response behavior of PSFT@CF was investigated under varying light intensities ranging from 50 to 100 mW/cm^2^. The results reveal that PSFT@CF exhibits excellent photothermal conversion properties (approximately 50 °C) even under low-power illumination (50 mW/cm^2^), as presented in Figure 3d,e. Meanwhile, the equilibrium temperature (T) of PSFT@CF displays an almost linear correlation with light intensity (L), as illustrated in Figure S7, thereby guaranteeing the controllability and operational safety of the superhydrophobic photothermal coated fabric in practical environments. The outstanding superhydrophobicity and superior photothermal conversion efficiency of PSFT@CF lay a robust fundamental basis for its practical utilization in anti-icing and de-icing applications. Figure 3f shows the temperature variation curve of PSFT@CF during repeated heating-cooling tests. The coated fabric exhibits consistently rapid photothermal response and high equilibrium temperatures in each cycle, demonstrating that the coating possesses excellent photothermal stability and satisfies the requirements for long-term use under sunlight conditions.

**Figure 4:**
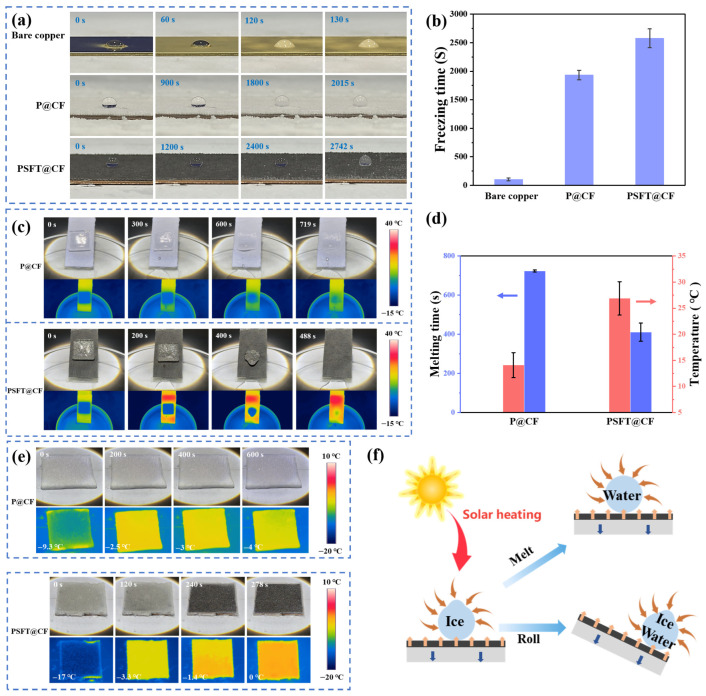
(**a**) Icing process of a water droplet on the bare copper, P@CF and PSFT@CF surface. (**b**) The freezing time of water droplets on the bare copper, P@CF and PSFT@CF. (**c**) Optical images and infrared thermal images of the photothermal de-icing process of P@CF and PSFT@CF under 100 mW/cm^2^ sun irradiation. (**d**) Ice melting off time (blue) and surface temperature (red) of the samples. (**e**) Static de-icing process of P@CF and PSFT@CF. (**f**) Schematic diagram of the anti-/de-icing mechanism.

Herein, the anti-icing and de-icing performances of PSFT@CF are investigated individually. As presented in Figure 4a, a bare copper plate, a P@CF-covered copper plate, and a PSFT@CF-covered copper plate are separately placed on a semiconductor cooling table with the temperature maintained at approximately −17 °C to evaluate the passive anti-icing performance of the samples. An equal volume of water droplets is dispensed onto the sample surfaces to monitor the freezing process. An instantaneous liquid-to-solid phase transition of water droplets on a bare copper plate is observed at around 120 s. In contrast, water droplets on P@CF-covered copper plate maintain a spherical shape and do not achieve complete freezing until approximately 2000 s. For water droplets on PSFT@CF-covered copper plate, the complete freezing time is further prolonged to about 2700 s, which is ascribed to its further optimized hierarchical micro/nanoscale rough structure. The superhydrophobic surface is capable of trapping abundant air to form an air cushion, thereby reducing the contact area and interfacial heat transfer between water and the superhydrophobic coating, which remarkably prolongs the freezing time of water droplets on superhydrophobic surfaces (Figure 4b).

Additionally, to investigate the photothermal active de-icing performance of PSFT@CF, the sample is placed on a semiconductor cooling table and tilted at a certain angle, with an ice cube positioned on its surface. The sample is then irradiated by simulated sunlight at 100 mW/cm^2^, and the melting and sliding detachments of the ice cube from the sample surface are recorded. As illustrated in Figure 4c, the ice cube on P@CF does not detach even after 719 s, leaving a large amount of water residue on the surface. In contrast, the ice cube on PSFT@CF melts completely and detaches smoothly at approximately 480 s, leaving no residual water on the sample surface. The presence of Fe^3+^-TA coordination complexes endows the coating with a rapid temperature increase under solar illumination (Figure 4d), thereby accelerating ice melting. Furthermore, the melted water can easily flow away from the superhydrophobic surface, which prevents re-icing on the photothermal surface under low-temperature conditions. The P@CF and PSFT@CF samples supported on copper plates are separately placed on a semiconductor cooling table for 1 h to ensure their surfaces are fully covered with ice. Subsequently, the samples are exposed to simulated sunlight at 100 mW/cm^2^, and the static photothermal de-icing performance is evaluated by monitoring the melting process of the surface ice layer. The results demonstrate that even after 600 s of solar irradiation, the ice layer on the P@CF surface shows no sign of melting, with its surface temperature being only −4 °C (Figure 4e). In comparison, the ice layer on the PSFT@CF surface begins to melt after merely 120 s and is almost completely converted into water within 278 s, accompanied by a surface temperature above 0 °C, which is higher than the freezing point of water. Figure 4f illustrates the anti-icing and de-icing mechanism of the superhydrophobic photothermal coating. Under specific light intensities, ice on the coating surface melts rapidly. Furthermore, the melted water is efficiently drained away from the superhydrophobic coating surface, thereby preventing the reformation of ice in low-temperature environments.

**Figure 5:**
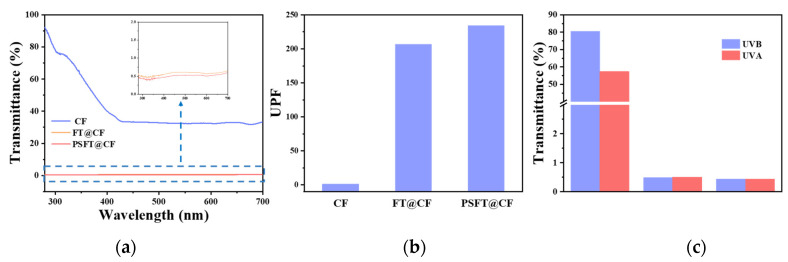
(**a**) UV-vis transmission spectra of the samples. (**b**) UPF level of the samples. (**c**) UV transmittance rates of the samples over UVA and UVB wavelengths.

Short-wavelength, high-energy ultraviolet (UV) light in solar radiation, including UVB (280–320 nm) and UVA (320–400 nm), can break covalent bonds in organic materials and cause irreversible damage to polymeric materials. Therefore, excellent UV shielding performance is critically important for solar-driven flexible anti-icing/de-icing materials. Previous studies have demonstrated that TA, as a polyphenolic material, exhibits outstanding UV shielding capability [38,39,40]. As shown in Figure 5a, after in situ growth of the TA-APTES coating followed by coordination with Fe^3+^ ions, the UV transmittance of FT@CF decreases sharply compared with that of CF. After coating with the PDMS/SiO_2_ layer, the UV transmittance of PSFT@CF shows no obvious change and remains at an extremely low level. To quantitatively evaluate the UV protection performance, the ultraviolet protection factor (UPF) values of various samples are calculated based on the transmittance spectra (Figure 5b). The UPF value of CF is merely 1.3, which fails to provide effective protection against UV damage. In contrast, the UPF values of FT@CF and PSFT@CF reach as high as 207 and 234, respectively, far exceeding the standard for UV-protective textiles (UPF > 50). It can be observed that PSFT@CF exhibits superior UV shielding performance in both the UVA and UVB regions, with transmittance values below 0.5% in both bands, further confirming its exceptional UV protection ability. The outstanding UV shielding property of the coating provides excellent protection for devices and extends the service life of materials and devices in outdoor environments, enabling efficient utilization of solar energy while avoiding the potential hazards of sunlight.

## 4. Conclusions

In summary, this study has presented a superhydrophobic photothermal coating with hierarchical rough structures based on metal–polyphenol coordination complex. The photothermal layer is fabricated via the in situ deposition of TA and APTES, followed by coordination with ferric ions, and the dip-coating of a PDMS/SiO_2_ layer is employed to optimize the micro–nano rough structures and achieve low-surface-energy modification. The coated fabric exhibits outstanding superhydrophobicity and photothermal conversion performance, demonstrating great application potential in passive anti-icing and active de-icing applications. Furthermore, the coating displays an ideal ultraviolet shielding property, which can provide sufficient protection for the coated fabric in solar-driven applications. Accordingly, this work offers a feasible strategy for the development of superhydrophobic coatings toward anti-icing/de-icing applications.

## Data Availability

Data are contained within the article or Supplementary Materials.

## Supplementary Materials

The following supporting information can be downloaded at https://www.mdpi.com/article/10.3390/polym18111286/s1. Figure S1: Reaction mechanism of metal-polyphenol coordination complex; Figure S2: The optical picture of CF, T@CF, FT@CF and PSFT@CF; Figure S3: SEM images of (a) and (a’) CF, (b) and (b’) T@CF, (c) and (c’) FT@CF; Figure S4: (a) SEM image of T@CF. (b) Particle size distribution of TA-APTES nanospheres.
