# Supplementary material for: In Situ Construction of Superhydrophobic Photothermal Coatings Based on Metal–Polyphenol Coordination Complex for Anti-/De-Icing Applications
Source: Polymers (Basel). 2026 May 24;18(11):1286. doi: 10.3390/polym18111286 (PMC13259266; doi:10.3390/polym18111286)
Supplement: Supplementary file 1 [file polymers-18-01286-s001.zip › polymers-4268181-supplementary.pdf]

polymers

MDPI

Article

In Situ Construction of Superhydrophobic Photothermal Coatings Based on Metal–Polyphenol Coordination Complex for Anti-/De-Icing Applications

Zhiheng Zhao [1,2], Buyu Luo [1], Guoliang Chen [1], Tianbao Zhao [1], Yifei Chen [3], Zhengping Zhao [3,*] and Baoshu Chen [1,*]

[1] School of Materials Science and Engineering, Xihua University, Chengdu 610039, China; zhaozh123@xhu.edu.cn (Z.Z.); xhucgl@icloud.com (G.C.); 0120140042@xhu.edu.cn (T.Z.)
[2] The State Key Laboratory of Polymer Materials Engineering, Polymer Research Institute of Sichuan University, Chengdu 610065, China
[3] Zhijiang College, Zhejiang University of Technology, Hangzhou 310014, China
* Correspondence: sjzhaolei@163.com (Z.Z.); cbs0926@xhu.edu.cn (B.C.)

Supplementary Materials

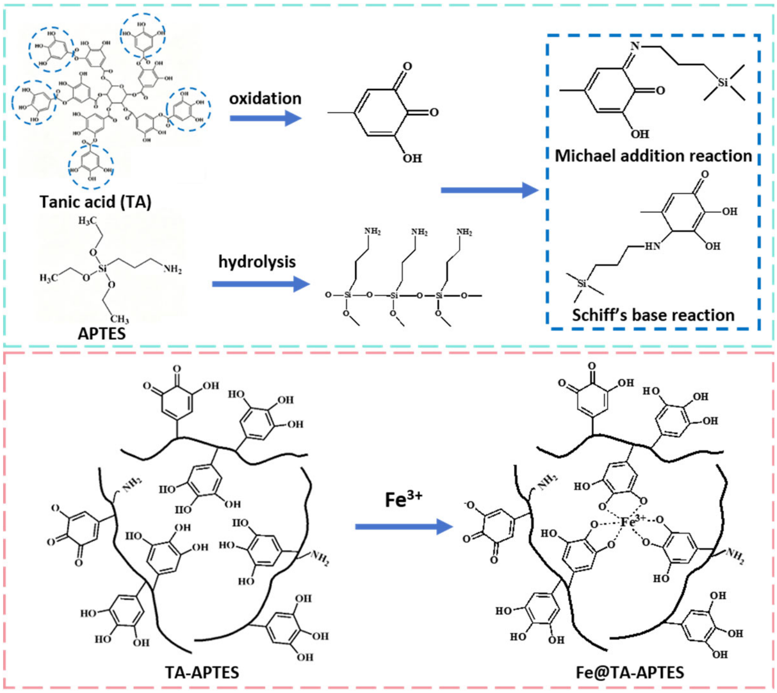

Figure S1. Reaction mechanism of metal-polyphenol coordination complex.

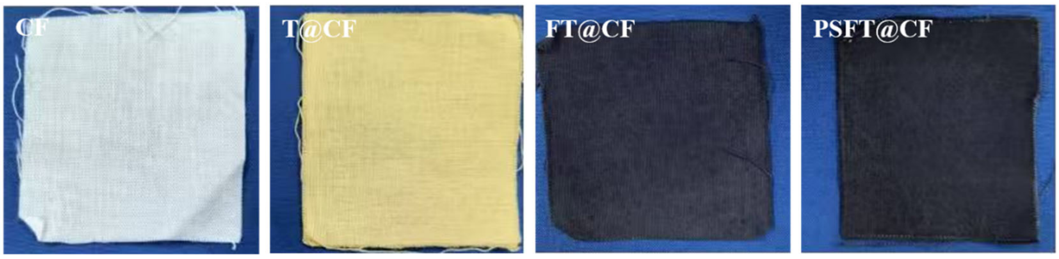

Figure S2. The optical picture of CF, T@CF, FT@CF and PSFT@CF.

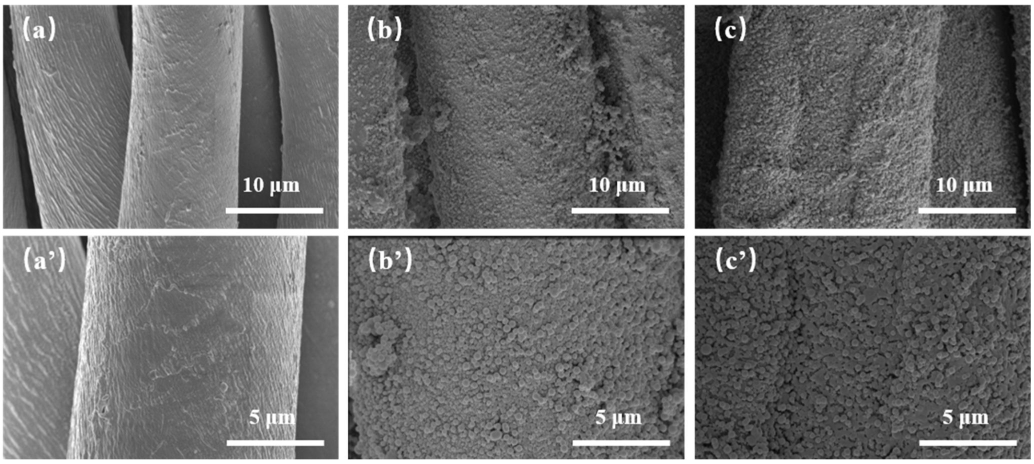

Figure S3. SEM images of (**a**) and (**a′**) CF, (**b**) and (**b′**) T@CF, (**c**) and (**c′**) FT@CF.

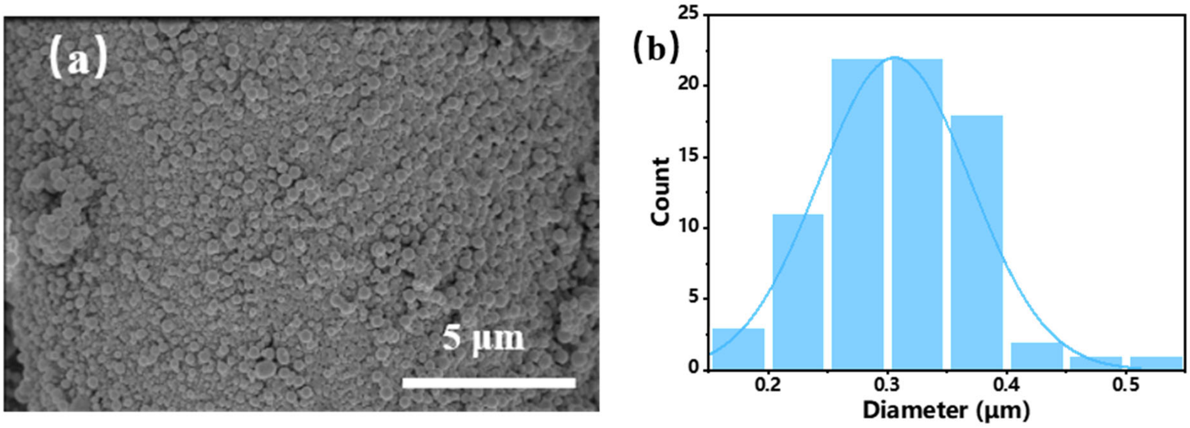

Figure S4. (**a**) SEM image of T@CF. (**b**) Particle size distribution of TA-APTES nanospheres.
